# Cardiac pacing in patients with hypertrophic obstructive cardiomyopathy

**DOI:** 10.21542/gcsp.2018.29

**Published:** 2018-08-12

**Authors:** José Maria Tolosana, Emilce Trucco

**Affiliations:** 1 Arrhythmias Section, Cardiology, Cardiovascular Institute, Hospital Clinic University, Barcelona, Spain; 2 Arrhythmias Section, Cardiology, Hospital Universitari Doctor Josep Trueta, Girona, Spain

## Introduction

About one-third of patients with hypertrophic cardiomyopathy (HCM) have resting left ventricular outflow tract obstruction (LVOTO). The presence of LVOTO in HCM facilitates the progression of heart failure and increases the risk of death^1,2^. LVOTO is defined as a peak instantaneous Doppler LV outflow tract gradient ≥30 mmHg, but the threshold for invasive treatment is usually considered to be ≥50 mm Hg^3^.

**Figure 1.**
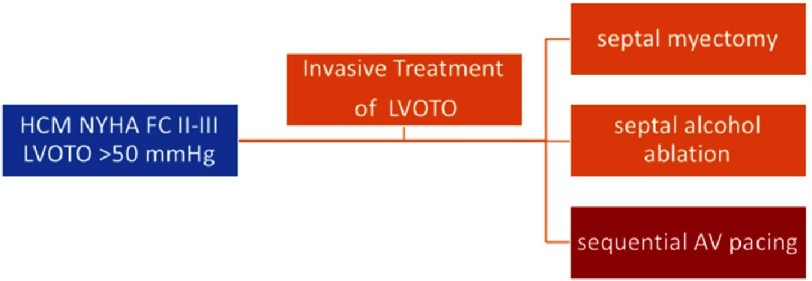
Invasive treatments to reduce LVOTO in symptomatic patients with an LVOTO gradient ≥50 mmHg.

Symptomatic treatment is directed towards decreasing the LVOT gradient. Medical therapy (beta-blockers, calcium channel blockers, and disopyramide) improves symptoms in the majority of patients. However, a percentage of patients do not improve with medical therapy and need an invasive treatment. Invasive treatment to reduce LVOTO should be considered in patients with an LVOTO gradient ≥50 mmHg, NYHA class III-IV and/or recurrent exertional syncope in spite of maximally tolerated drug therapy^3^. There are different strategies of invasive treatment of LVOTO: 1) septal myectomy; 2) septal alcohol ablation (SAA) and 3) sequential DDD-AV pacing (Figure 1). In this review we describe the indications and the evidence of sequential DDD-AV pacing and the beneficial effects of biventricular pacing to reduce the gradient of LVOTO in patients with HCM.

## Sequential DDD-AV right ventricular pacing

In medically refractory symptomatic patients with hypertrophic obstructive cardiomyopathy (HOCM) who are suboptimal candidates for septal reduction therapy, DDD pacing with a short AV delay is an alternative strategy. The mechanism of action of AV sequential pacing is not completely elucidated. Hypotheses to explain the beneficial effects include: 1) negative inotropic effect and reduced hypercontractility of the LV; 2) asynchronous septal activation and delayed septal thickening; 3) limitation of abnormal mitral valve motion; 4) interactions with LV filling, and 5) ventricular remodelling^4^.

Three small, randomized, placebo-controlled studies of dual chamber pacing and several long-term observational studies have reported reductions in LV outflow tract gradients but a variable improvement in symptoms and quality of life^5–7^. A European multicentre double-blind cross-over trial (the PIC study) in 83 patients of active vs. inactive short AV delay pacing demonstrated that active pacing was associated with a significantly lower outflow gradient, reduction in symptoms, and improved NYHA class and quality of life, with effect persisting at 1 year. Surprisingly, even pacemaker implantation without activation of AV sequential pacing lowered the LVOTO but subsequent activation of pacing in the same patients showed further reductions. Exercise tolerance was only improved in those with severe baseline impairment^5^.

Another randomized, double-blind cross-over study (the MPATHY study) with 48 patients confirmed significant benefit of pacing therapy on outflow gradient and quality of life score but without improved exercise capacity. There were some responders, but they were significantly older (69 ± 4 years) than non-responders (51 ± 16 years) and had a lower baseline exercise time and peak VO2 than non-responders. The authors concluded that DDD pacing might be an option for patients >65 years if they reject other therapeutic options^6^.

Finally, in a Spanish study with 82 patients, sequential pacing in selected patients with HOCM improved functional class and reduced dynamic gradient and mitral regurgitation immediately after pacemaker implantation and at final follow-up. Prolonged ventricular pacing appeared not to have negative effects on systolic or diastolic function^7^.

These studies do not provide sufficient data to compare results on all-cause mortality, cost effectiveness, exercise capacity, quality of life, and peak O_2_ consumption^8^. Furthermore, one study has directly compared SAA with pacing and demonstrated superior gradient reduction with ablation^9^.

The conflicting data on both gradient reduction and functional capacity improvement mean that current clinical practice guidelines recommended sequential DDD pacing only for symptomatic patients who have contraindications for SAA or myectomy or are at high risk of developing heart block following SAA or myectomy. Sequential DDD pacing may also be an option for patients with resting or provocable LVOTO ≥50 mmHg, sinus rhythm and drug refractory symptoms, in whom there is an indication for an ICD or pacing for bradyarrhythmia^3^.

Several studies have suggested that the effectiveness of pacing can greatly improved by individual optimization of pacemaker settings. Pacing parameters should be optimized to achieve maximum pre-excitation of the RV apex with minimal compromise of LV filling (too short a paced AV interval results in hemodynamic deterioration and too long a atrioventricular interval without complete pre-excitation of the ventricle results in an inadequate response). Atrioventricular nodal ablation or modification has been advocated as a method for achieving optimal AV programming in some patients with a very short P-R interval^10^.

### Sequential biventricular pacing in HOCM to reduce LVOTO

**Figure 2.**
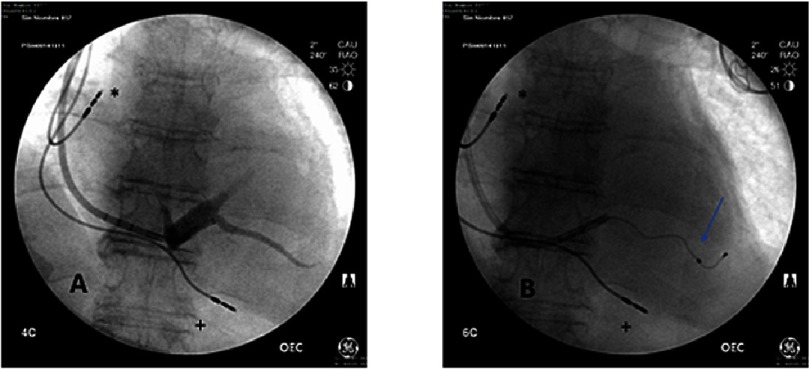
Implant of a left ventricular lead in a lateral vein thorough the coronary sinus. Postero anterior view. (A) Cannulation of the CS and lateral vein; (B) Placement of left ventricular lead. * Atrial lead; + Right ventricular lead; **Blue arrow**: Left ventricular lead.

Recent data show that atrial synchronous left ventricular (LV) or biventricular (BiV) pacing might further reduce the LVOT pressure gradient and improve symptoms in patients with HOCM and LVOT obstruction^11–16^ (Figure 2). Data obtained from small and observational studies showed that LVOT gradient reduction obtained with LV or BiV pacing was superior to conventional RV apex pacing. Furthermore, LV or BiV pacing improves the symptoms and the functional capacity in a large proportion of HOCM patients not suitable for other invasive treatments like myectomy or alcohol septal ablation. (Table 1)

**Table 1:** Studies comparing RV pacing vs LV/BiV pacingand clinical and LVOT reduction between both strategies.

Study	N	Basal NYHA	Follow-up NYHA	Basalc 6MWDT	Follow-up 6MWDT	Basal LVOT	Acute RVp LVOT	Acute LV/BIVLVOT	Chronic LV/BIVLVOT
Berruezo A et al.^15^	9	3.2 ± 0.4	1.4 ± 0.5	349 ± 116	517 ± 206	74 ± 23	69 ± 25	50 ± 27	28 ± 17
Rinaldi CA et al.^16^	8	NA	NA	NA	NA	67 ± 25	41 ± 15	25 ± 15	NA
Lenarczyk R et al.^14^	8	II	I	345	446	64	NA	NA	33

**Notes.**

6MWDT 6 mean walk distance test LVOT Left ventricular outflow tract RVp Right ventricular pacing LV/BIV Left ventricular/biventricular pacing

Berruezo et al.^15^, in a pilot study, demonstrated that BiV or LV pacing were superior to RV pacing and was the best configuration for acute LVOT gradient reduction in selected patients with HOCM and sinus rhythm. Moreover, the best possible configuration obtained with BiV or LV pacing was associated with a progressive reduction of LV mass (reverse remodelling) and a significant improvement of symptoms, functional capacity, and quality of life. The magnitude of improvement in NYHA class, quality of life and functional capacity were superior to other long-term observational or randomized studies on DDD RV pacing in HOCM^5,6,10,17^.

### Biventricular pacing optimization in HOCM

All studies^14–16^ that have shown the beneficial effects of BiV pacing in HOCM optimized the A-V and the V-V intervals by echo after the implant. The optimal A-V interval was considered as the shortest AV interval that did not truncate the A wave on pulsed Doppler of the mitral inflow^18^.

With the optimum AV interval set, LVOTG reduction was tested consecutively during right ventricle (RV) pacing, LV pacing, or biventricular pacing (RV pre-activation [30 ms], simultaneous biventricular pacing VV, or LV pre-activation [30 ms]). The optimal VV interval was selected on the basis of maximum reduction of LVOTG after a 5-minute equilibrium phase for each set up^15^.

### Mechanism of LVOT gradient reduction in HOCM by LV or BiV pacing

Unlike cardiac resynchronization therapy in dilated cardiomyopathies, the objective of BiV-pacing in patients with HOCM is to dyssynchronize the ventricular contraction and change the pattern of LV activation. LV/BiV pacing might be able to induce a supplementary reduction in LVOT gradient by an alteration in the contraction of a larger area of the LV. The reversed LV depolarization sequence [caused by pre-excitation of the LV posterolateral/lateral wall during LV/BiV pacing] may activate the longitudinally oriented epicardial fibers earlier, advancing the lateral wall longitudinal displacement with regard to interventricular septal longitudinal displacement^19^.

Giraldeu G et al.^20^ analysed by echo the LV mechanical displacement of the patients with HOCM who were consider responders to BiV pacing. A responder was defined as one who had a follow-up gradient <50 mmHg and had a reduction in LVOT pressure gradient of at least 50% from baseline. Patients with HOCM who obtained a better response to BiV pacing had an inversion of the wall motion timing with an earlier displacement of the lateral wall and a delay of the septal wall. In patients who did not respond to the therapy this phenomenon was not observed. Therefore, the mechanical dyssynchrony induced by LV or BiV pacing which inverted the timing of LV wall activation plays an important role in the reduction of LVOT obstruction.

By changing the timing of LV activation and inverting the LV wall displacement, BiV pacing might create changes in the diameter of the LVOT and geometry of the mitral valve apparatus resulting in the reduction of LVOT obstruction observed at short time. At long term follow-up, the LV reverse remodelling observed in these patients may play an additional role by the reduction of the septal and posterior wall thickness.

### The importance of avoiding fusion between intrinsic and paced rhythm in patients with HOCM treated with biventricular pacing

The optimal AV delay is defined as the shortest interval able to produce full ventricular capture by pacing (without fusion with intrinsic QRS) with no impairment of diastolic filling. Programming an optimal AV delay in practice, may be difficult since the AV-delay must be short enough to fully capture the LV and exclude fusion, but on the other hand, long enough in order to allow complete LV filling. Atrioventricular node ablation (AVNA) could be necessary to obtain complete ventricular pre-excitation without impairment of diastolic filling in these patients.^21^

BiV pacing changes the timing of LV activation, which delays contraction of the basal interventricular septum and therefore increases the systolic dimension of the LVOT, with secondary reduction in systolic anterior motion of the mitral valve. However, to be effective, pacing must induce a full ventricular capture (depolarization only caused by pacing) of the LV, because the septum is the first part of the myocardium to be depolarized by intrinsic conduction. AVN ablation ensures this mechanism, because it prevents the intrinsic depolarization of the interventricular septum.

In the series of Berruezo et al. the mean LVOT gradient decreased from 80 ± 25 to 10 ± 17 mmHg at end follow-up in patients without fusion while there were no significant changes (from 81 ± 25 to 58 ± 32 mmHg) in patients with fusion. Patients with fusion also did not improve in symptoms. The observations of the present study confirm that the presence of fusion minimizes the benefit of pacing on LVOTG reduction, as well as on clinical improvement^22^.

**Figure 3.**
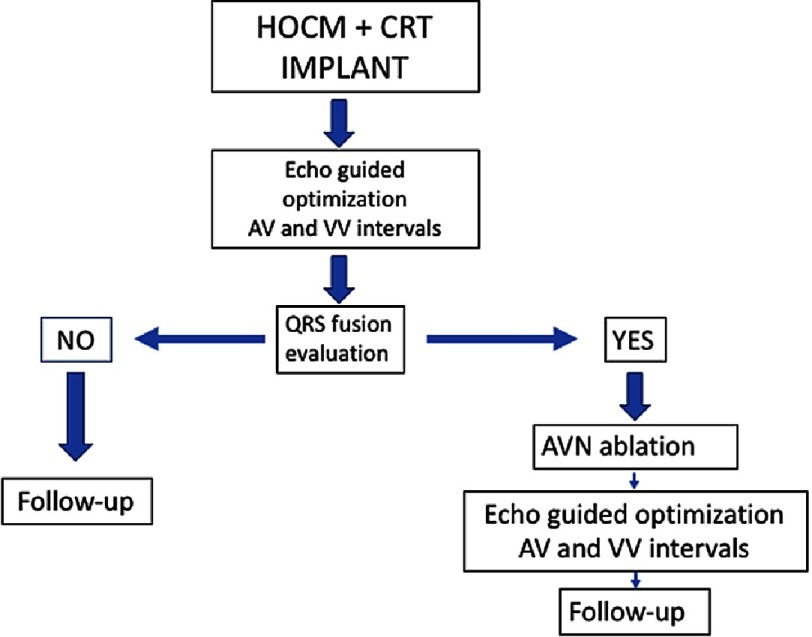
Protocol to follow in patients with HOCM after CRT implant, depending the presence of fusion between the intrinsic and paced QRS rhythm or not. HOCM, Hypertrophic obstructive cardiomyopathy; AVN, Atrioventricular node; AV, Atrio ventricular; VV, Interventricular intervals.

After echocardiographic AV optimization, ECG fusion is present in approximately 60% of patients with AS-BiVP and nullifies the benefit of pacing. In patients with ECG fusion, AVNA further reduces LVOTG. Atrial synchronous biventricular pacing (AS-BiVP), which ensures no ECG fusion by means of AVNA when needed, appears to be the optimal pacing mode for gradient reduction in HOCM patients and significantly improves functional capacity and quality of life (Figure 3).

### How to detect fusion between intrinsic and BiV pacing rhythm

**Figure 4.**
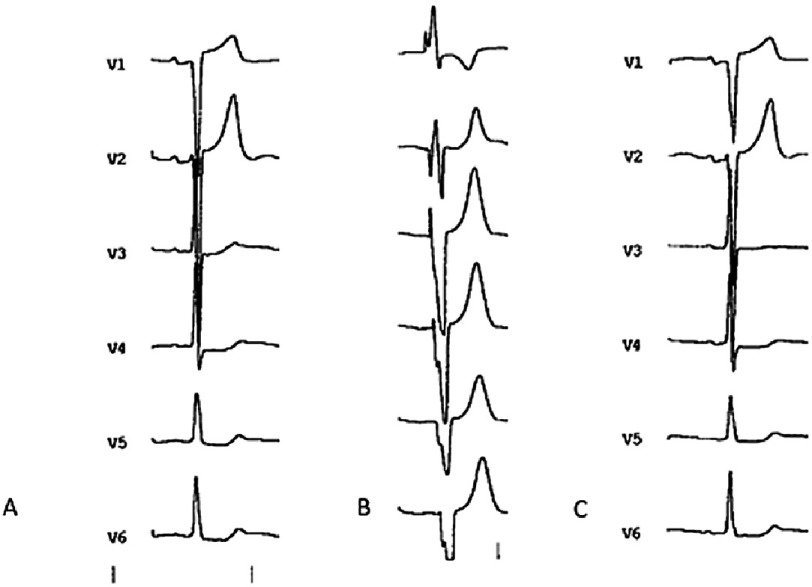
QRS morphology: (A) intrinsic; (B) VVI BiV paced; (C) Paced after CRT optimization. Fusion between intrinsic and optimized paced BiV QRS is observed.

A basal 12-lead ECG is obtained to record the intrinsic PR interval and QRS morphology. After CRT implant, the device is programmed to VVI, BiV pacing with optimal VV interval defined by echocardiography, and pacing rate 10 bpm higher than the intrinsic heart rate. Then, a 12-lead ECG is obtained to record the morphology of the paced QRS without fusion. Finally, the device is programmed to optimized DDD, BiV pacing, with the optimal sensed AV and VV intervals defined by echocardiography. Then, a 12-lead ECG is obtained. Fusion between intrinsic LV depolarization and paced QRS will be considered when changes in duration, morphology or axis are detected in any of the 12 leads surface ECG of VVI paced QRS and QRS obtained after programming the device according echo optimization (Figure 4).

A prospective, randomized, single-blinded, multicenter study named TRICHAMPION is designed to investigate the benefit of optimized atrial synchronous biventricular pacing in absence of QRS fusion in highly symptomatic HOCM patients with severe LVOT obstruction who are not candidates for ablative therapies. The results of this trial may answer several questions about the role and the optimization of A-V biventricular sequential pacing in these patients^23^.

## Conclusion

In selected patients with HOCM, and non-candidates for myectomy or SAA, sequential A-V BiV pacing is feasible and usually the best configuration for LVOT gradient reduction. Overall, BiV pacing in patients with HOCM significantly and progressively improves functional capacity, quality of life and may also induce LV reverse remodeling.

It should be highlighted that the benefits of sequential DDD-BiV pacing in HOCM appear when fusion between intrinsic and paced rhythm does not exist after the device programming optimization, therefore a high percentage of these patients will required AVN ablation.
